# Genome-wide multi-trait analysis of irritable bowel syndrome and related mental conditions identifies 38 new independent variants

**DOI:** 10.1186/s12967-023-04107-5

**Published:** 2023-04-21

**Authors:** Silvia Alemany, María Soler-Artigas, Judit Cabana-Domínguez, Dana Fakhreddine, Natalia Llonga, Laura Vilar-Ribó, Amanda Rodríguez-Urrutia, Judit Palacio, Ana María González-Castro, Beatriz Lobo, Carmen Alonso-Cotoner, Magnus Simrén, Javier Santos, Josep Antoni Ramos-Quiroga, Marta Ribasés

**Affiliations:** 1 Psychiatric Genetics Unit, Group of Psychiatry Mental Health and Addiction, Vall d’Hebron Research Institute (VHIR), Universitat Autònoma de Barcelona, Passeig Vall d’Hebron, 119-129, 08035 Barcelona, Spain; 2 Department of Mental Health, Hospital Universitari Vall d’Hebron, Barcelona, Spain; 3 Biomedical Network Research Centre On Mental Health (CIBERSAM), Instituto de Salud Carlos III, Madrid, Spain; 4 Department of Genetics, Microbiology, and Statistics, Faculty of Biology, Universitat de Barcelona, Barcelona, Spain; 5 Department of Psychiatry and Forensic Medicine, Universitat Autònoma de Barcelona, Barcelona, Spain; 6 Laboratory of Neuro-Immuno-Gastroenterology, Digestive System Research Unit, Vall d’Hebron Institut de Recerca (VHIR), Vall d’Hebron Hospital Universitari, Barcelona, Spain; 7 Department of Gastroenterology, Vall d’Hebron Barcelona Hospital Campus, Vall d’Hebron Hospital Universitari, Passeig Vall d’Hebron 119-129, 08035 Barcelona, Spain; 8 Department of Medicine, Universitat Autònoma de Barcelona, Barcelona, Spain; 9 Centro de Investigación Biomédica en Red de Enfermedades Hepáticas y Digestivas (CIBERHED), Instituto de Salud Carlos III, Madrid, Spain; 10 Department of Molecular and Clinical Medicine, Institute of Medicine, Sahlgrenska Academy, University of Gothenburg, Gothenburg, Sweden; 11 Centre for Functional GI and Motility Disorders, University of North Carolina, Chapel Hill, NC USA

**Keywords:** Irritable bowel syndrome (IBS), Neuroticism, Depression, Anxiety, Multi-trait genome-wide association study (MTAG)

## Abstract

**Background:**

Irritable bowel syndrome (IBS) is a chronic disorder of gut-brain interaction frequently accompanied by mental conditions, including depression and anxiety. Despite showing substantial heritability and being partly determined by a genetic component, the genetic underpinnings explaining the high rates of comorbidity remain largely unclear and there are no conclusive data on the temporal relationship between them. Exploring the overlapping genetic architecture between IBS and mental conditions may help to identify novel genetic loci and biological mechanisms underlying IBS and causal relationships between them.

**Methods:**

We quantified the genetic overlap between IBS, neuroticism, depression and anxiety, conducted a multi-trait genome-wide association study (GWAS) considering these traits and investigated causal relationships between them by using the largest GWAS to date.

**Results:**

IBS showed to be a highly polygenic disorder with extensive genetic sharing with mental conditions. Multi-trait analysis of IBS and neuroticism, depression and anxiety identified 42 genome-wide significant variants for IBS, of which 38 are novel. Fine-mapping risk loci highlighted 289 genes enriched in genes upregulated during early embryonic brain development and gene-sets related with psychiatric, digestive and autoimmune disorders. IBS-associated genes were enriched for target genes of anti-inflammatory and antirheumatic drugs, anesthetics and opioid dependence pharmacological treatment. Mendelian-randomization analysis accounting for correlated pleiotropy identified bidirectional causal effects between IBS and neuroticism and depression and causal effects of the genetic liability of IBS on anxiety.

**Conclusions:**

These findings provide evidence of the polygenic architecture of IBS, identify novel genome-wide significant variants for IBS and extend previous knowledge on the genetic overlap and relationship between gastrointestinal and mental disorders.

**Supplementary Information:**

The online version contains supplementary material available at 10.1186/s12967-023-04107-5.

## Introduction

Irritable bowel syndrome (IBS) is one of the most prevalent disorders of gut-brain interaction with a population lifetime risk of 11% [1] and a point prevalence of 4.1% according to the strict Rome IV criteria [2]. IBS research is extremely challenging due to the multifactorial etiology of the disease and the heterogeneity of patients, who present high comorbidity rates for mental disorders, particularly, anxiety and depression, which impacts negatively on the patients’ quality of life [1, 3, 4].

A recent systematic review revealed that the prevalence of anxiety and depression symptoms among IBS patients is 39.1% and 28.8%, respectively [5]. In addition, IBS has been associated with more severe depressive symptoms compared to healthy controls and, when co-existing with psychiatric disorders, gastrointestinal symptoms are more severe and disabling [6–11]. This close association between IBS, anxiety and depression is also supported by neuroimaging studies and might be related to the bi-directional communication between the brain and the digestive system, termed the brain-gut-axis, which occurs through microbiota, neural, neuroimmune and neuroendocrine pathways [12–14]. This idea agrees with evidence indicating that psychiatric interventions, including antidepressants or cognitive-behavioral therapy, improve IBS patients functioning and suggests that common pathophysiological mechanisms may be underlying these conditions [15].

IBS, anxiety and depression are partly determined by a genetic component and show substantial heritability ranging from 6% for IBS to 30%-50% for anxiety and depression [16–18]. The largest genome-wide association study (GWAS) on IBS conducted to date included 53,400 cases and 433,201 controls and identified six genome-wide significant single nucleotide polymorphisms (SNPs) [18] which represents an improvement over the previous study, identifying four independent genome-wide significant SNPs [19]. Interestingly, among 173 traits, three mental conditions (neuroticism, depression and anxiety) were the most genetically correlated traits with IBS [18]. Despite these strong genetic correlations, the genetic underpinnings explaining the high rates of comorbidity between IBS and mental conditions remain largely unclear and there are no conclusive data on the temporal and causal relationship between them [18, 19].

In the present study we investigated the shared genetic architecture and the nature of the relationship between IBS and three highly genetically correlated conditions (neuroticism, depression and anxiety) using summary statistics of the largest GWAS datasets available so far by (i) estimating the genetic correlation and overlap between them, (ii) conducting a Multi-Trait Analysis of GWAS (MTAG) to identify novel genetic loci for IBS and (iii) performing downstream analyses to explore the overlaping genetic basis with other disorders and traits as well as causal relationships between them.

## Materials and methods

### Samples

We used publicly available SNP-level GWAS summary statistics for IBS [18], neuroticism [20], depression [21] and anxiety (Table 1). For further details see Additional file 1: Note 1.

**Table 1:** Summary of the GWAS datasets used in the current study

Phenotype	N cases	N controls	N total	N effective ^a^	GWAS genome-wide significant SNPs^b^	References
IBS	53,400	433,201	486,601	190,159	6	[18]
Neuroticism	–	–	390,278^c^	390,278	136^d^	[20]
Depression	170,756	329,443	500,199^c^	449,856	102^d^	[21]
Anxiety nerves or GAD	16,730	101,021	117,751	57,412	1	UKBB phenotype code: 20544_15

*GAD* generalized anxiety disorder; *UKBB* UK Biobank

^a^N effective sample sizes were calculated following the equation: Neff = 4/(1/Ncases + 1/Ncontrols)

^b^Number of genome-wide significant independent SNPs

^c^Sample size excluding the 23andMe cohort

^d^Genome-wide significant SNPs including the 23andMe cohort

### SNP-based heritability, genetic correlation and overlap

SNP heritability (*h*^*2*^_SNP_) and pair-wise genetic correlation between IBS and each mental condition was calculated using linkage disequilibrium score regression (LDSC) analysis [22]. Conversion of *h*^*2*^_SNP_ estimates from observed to liability scale was done using a population prevalence of 11%, 25%, 30% and 14% for IBS, neuroticism, depression and anxiety, respectively. Polygenic overlap between IBS and each mental condition was quantified using MiXeR [23]. MiXeR caclulates the number of trait-influencing SNPs for each trait (univariate model) and for both traits (bivariate model) and the proportion of variants with concordant direction of effects for both traits. The proportion of SNPs shared by two traits is indicated by the Dice coefficient. Model fit was assessed using the Akaike Information Criterion (AIC). For further details see Additional file 1: Note 2.

### Multi-trait analysis of GWAS (MTAG)

To identify new loci for IBS, SNP-level GWAS for IBS, neuroticism, depression and anxiety were meta-analyzed using MTAG [24]. MTAG estimates trait-specific effects from GWAS summary statistics of several traits genetically correlated while accounting for sample overlap across the discovery samples [24]. To discard inflation in our results we calculated the max-false discovery rate (max-FDR) using default settings as previously described [24, 25]. The LDSC intercept was used to quantify inflation resulting from confounding bias [22].

Independent SNPs from MTAG-IBS results (P-value < 5E-08) were identified through clumping (r^2^ = 0.05, kb = 5000) using the 1000 Genomes Project Phase 3 European reference panel (http://www.internationalgenome.org/) and PLINK1.09 as described by Eijsbouts et al. [18]. We defined loci as a 1Mb region centered around the most significant variant (lead variant) and we carried out conditional analyses to confirm independence between lead and any other variant identified in the clumping step (secondary variants) within each locus (i.e. within 1Mb and r^2^ < 0.05) using COJO implemented in Genome-wide Complex Trait Analysis (GCTA) [26]. For further details on conditional analysis see Additional file 1: Note 3.

### Credible variants and functional annotation

Sets of credible variants (credible-sets) were identified by fine-mapping the independent lead SNPs of MTAG-IBS using three different tools, FINEMAP 1.3.1 [27], PAINTOR v3.0 [28] and CAVIARBF v.0.2.1 [29] following the pipeline available elsewhere [30]. Variants located in a region of 1Mb around the lead SNPs were included in the analysis and we assumed that there was only one causal variant per locus. We used the recommended parameters of each tool and only variants identified by all three methods were considered. Functional annotation of the credible variants was conducted using FUMA [31]. For further details see Additional file 1: Note 4.

### Gene-based and gene-set analyses of MTAG-IBS results

Gene-based and gene-set analyses of MTAG-IBS associated SNPs were performed using MAGMA v1.08 [32] implemented in FUMA [31]. Tissue specific gene expression was explored using MAGMA gene-property analysis of expression data from GTEx v8 and BrainSpan available in FUMA (databases detailed in Additional file 1: Note 5). All gene sets were obtained from the Molecular Signatures Database (MSigDB v6.2) and included GO, KEGG, BIOCARTA and Reactome representing a total of 11,960 gene sets. The Bonferroni-corrected significance threshold for gene-based analysis was 0.05/18135 genes = 2.7571E-06 and for gene-set analysis was 0.05/11960 gene sets = 4.18E-06.

### Drug target identification

To explore whether finemapped genes related with IBS were enriched for target genes of drugs (druggable genes) we performed enrichment analysis based on information from the PharmGKB using WebGestAlt [33]. Identified drugs were classified according to available information from the Anatomical Therapeutic Chemical (ATC) classification system.

### Partitioned heritability and genetic correlations

We partitioned *h*^*2*^_SNP_ of MTAG-IBS results by functional annotation categories using stratified LDSC [34]. We calculated whether any of the 28 specific genomic categories included in the analysis was enriched for variants that contribute to *h*^*2*^_SNP_. Annotations for these functional genomic categories (e.g. coding or regulatory regions) were obtained from LDSC website (https://github.com/bulik/ldsc/wiki/Partitioned-Heritability) and included coding; intron; promoter; 3′5′ untranslated region; digital genomic footprint; transcription factor binding site; chromHMM and Segway annotations for six cell lines; DNase I hypersensitivity sites; H3K4me1, H3K4me3 and H3K9ac marks; two sets of H3K27ac marks; super-enhancers; conserved regions in mammals; and FANTOM5 enhancers (further details in Additional file 1: Note 6). We focused on categories extended by 500 bp in either direction. Enrichment/depletion of heritability in each category is calculated as the proportion of heritability attributable to SNPs in the specified category divided by the proportion of total SNPs annotated to that category. The Bonferroni-corrected significance threshold was 0.05/28 annotations = 0.0018.

We explored genetic correlations between our MTAG-IBS results and gastrointestinal, immunological and psychiatric disorders using LDSC analysis [22]. We selected all GWAS summary statistics of gastrointestinal/abdominal, immunological/systemic (UK Biobank: 21 phenotypes) and psychiatric disorders (PGC: 7 phenotypes) available in the MR-Base database (Additional file 3: Table S14) [35]. We used GWAS summary statistics including both males and females of European ancestry. If several GWAS were available for the same disorder, we chose the study with the largest effective sample size (N effective = 4/(1/Ncases + 1/Ncontrols)). The Bonferroni-corrected significance threshold used was 0.05/28 traits = 0.0018.

### Causal analysis using summary effect estimates (CAUSE)

Causal relationships between IBS and correlated traits were assessed considering independent variants (r^2^ = 0.05; kb = 5000) associated with the exposure with *P* < 1.0E-03 using CAUSE [36]. Bidirectional relationships were tested considering IBS as exposure and depression, anxiety or neuroticism as outcomes and vice-versa. Given that standard errors, required by CAUSE, were not available from the largest study on neuroticism to date [37], we used the GWAS dataset on neuroticism by Luciano et al. in 329,821 subjects as an alternative [38]. The strengths of CAUSE involve accounting for correlated horizontal pleiotropic effects (i.e. when a variant affects the outcome and the mediator through shared heritable factors) and using a less stringent significance threshold (P < 1.0E-3) allowing the incorporation of more variants to the analyses. CAUSE compares two nested models, a sharing and a causal model. Both models allow for horizontal pleiotropy (correlated pleiotropy (eta)) but only the casual model includes a causal effect parameter (gamma). The sharing and the causal model are compared against a null model and against each other. Model comparisons are carried out using the expected log pointwise posterior density (ELPD), a Bayesian model comparison approach that estimates how well the posterior distributions of a particular model are expected to predict a new set data. When P < 0.05 the second model fits the data better than the first model. There is evidence of causal effects when the causal model represents a significant improvement in the model fit of the sharing model.

For further details see Additional file 1: Note 7.

## Results

### SNP-based heritability, genetic correlation and overlap

The latest GWAS on IBS [18], neuroticism [20], depression [21] and anxiety used herein are summarized in Table 1 and Additional file 1: Note 1. The estimated SNP heritability (*h*^*2*^_SNP_) was 6.9% (SE = 0.004) for IBS, 14.6% (SE = 0.005) for neuroticism, 9.9% (SE = 0.004) for depression and 8.3% (SE = 0.011) for anxiety (Table 2). We found evidence of strong genetic correlation between IBS and all three mental conditions, ranging from 53 to 68% (Table 2). Univariate MiXeR analysis revealed that IBS and neuroticism were highly polygenic, with around twelve thousand variants explaining 90% of SNP heritability (12,438 variants for IBS and 12,308 for neuroticism; Additional file 3:Table S1a). Bivariate MiXeR analysis showed that the majority of the variants influencing IBS were shared with neuroticism (10,793 (SE = 1094) out of 12,438 (SE = 1305) variants, Dice coefficient = 0.87), with a high proportion of variants being concordant (71%) (Additional file 3: Table S1a and Additional file 2: Figure S1). Unfortunately, MiXeR was unable to accurately quantify the genetic overlap between IBS and depression or anxiety according to the Akaike Information Criterion (AIC; Additional file 3: Table S1b).

**Table 2:** Genetic correlation estimates for IBS and neuroticism, depression and anxiety using Linkage Disequilibrium Score Regression (LDSC)

Trait 1	Trait 2	Genetic Correlation	SE	Z	*P*-value	Intercept (SE)	Trait 1	Trait 2
							*h*^*2*^ (SE)	*h*^*2*^ (SE)
IBS	Neuroticism	0.526	0.027	19.298	5.54E-83	1.013 (0.013)	0.069 (0.004)	0.146 (0.005)
IBS	Depression	0.587	0.026	22.714	3.23E-114	0.992 (0.01)	0.069 (0.004)	0.099 (0.004)
IBS	Anxiety	0.677	0.065	10.360	3.75E-25	0.999 (0.74)	0.068 (0.004)	0.083 (0.011)

*SE*, standard error; *h*^*2*^ heritability

### Multi-trait analysis of GWAS (MTAG)

To identify novel loci for IBS, we combined the summary statistics from the GWAS on IBS, neuroticism, depression and anxiety using MTAG, increasing the estimated effective sample size from 486,601 in the original IBS dataset to 887,490. The max-FDR of MTAG-IBS analysis was low (0.020) suggesting no inflation, consistent with the similar mean chi-square values for the different GWAS, ranging from 1.08 for anxiety to 1.69 for neuroticism. There was no evidence of residual stratification or confounding leading to an inflation of test statistics (LD Score regression intercept = 0.857, SE = 0.009, See Additional file 2: Figure S2).

**Fig. 1:**
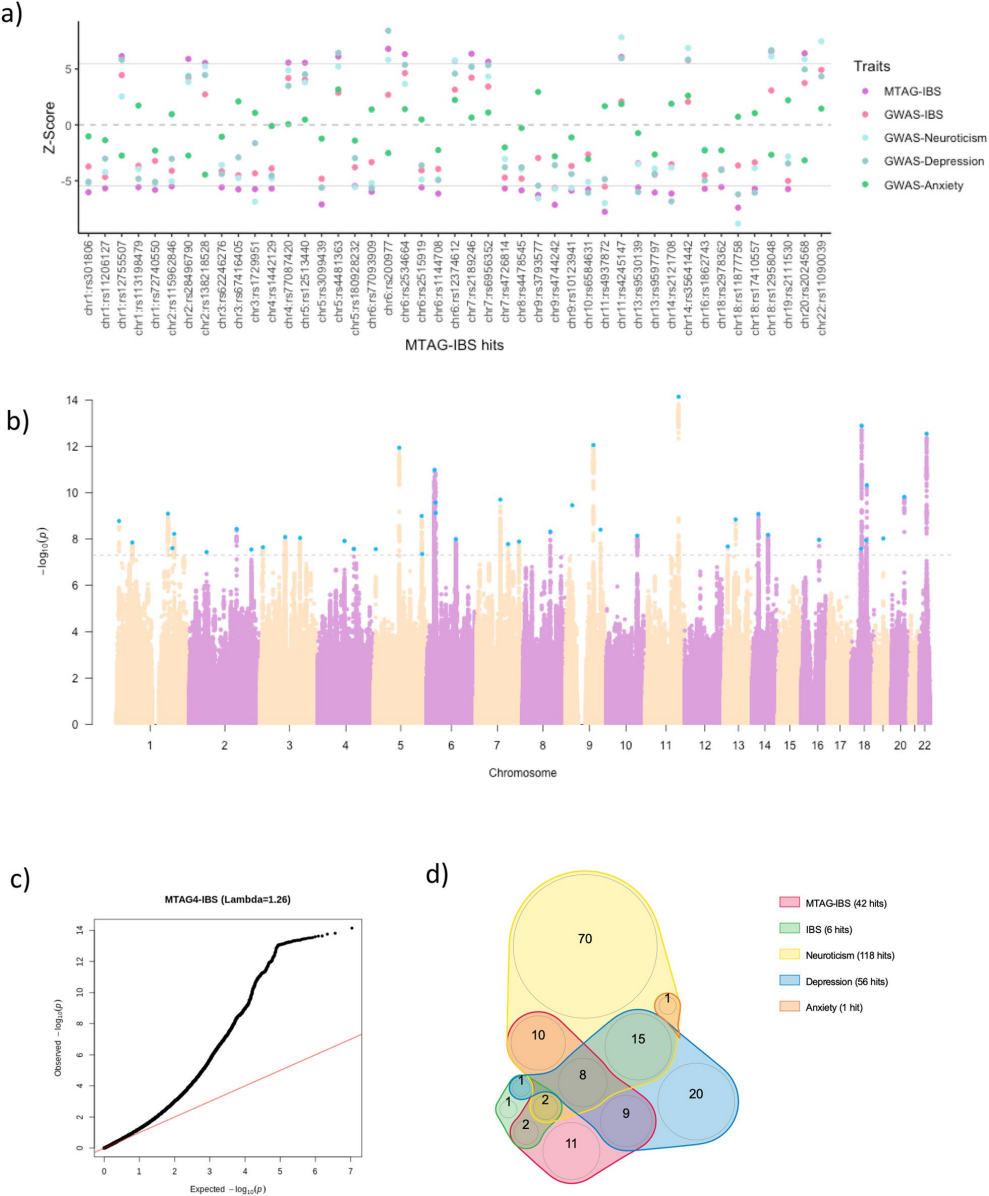
MTAG results of IBS and overlap with previous GWAS on IBS, neuroticism, depression and anxiety. **a** Z-scores of MTAG-IBS and original GWAS on IBS, neuroticism, depression and anxiety for each of the independent lead SNPs (n = 42) found in MTAG-IBS results. Dotted grey line indicates 0 Z-score and solid grey lines indicate statistical significance at P < 5-E08. **b** Manhattan plot of the MTAG-IBS results. Dotted grey line indicates statistical significance at P < 5-E08. **c** QQ plot of the MTAG-IBS results. **d** Venn diagram depicting overlap among MTAG-IBS independent lead SNPs and genome-wide significant SNPs in the original GWAS

After MTAG analysis, the number of genome-wide significant SNPs for IBS increased from six in the original GWAS to 42 independent SNPs in 37 loci (r^2^ < 0.05 between variants within each locus defined as regions of 1Mb) in the current study (Fig. 1, Additional file 2: Figure S3, Additional file 3: Table S2, S3). Five loci in chromosomes 5, 6, 11 and 18 (there were 2 loci in chromosome 18) had one or more secondary variants (i.e. in each locus there were more than one independent genome-wide significant variants). After conditional analysis to confirm independence, secondary variants remained significant in the loci in chromosome 6 (4 secondary variants), 5 (1 secondary variant) and 18 (1 secondary lead variant). The secondary variant in chromosome 11 was no longer significant after conditional analysis leaving only the lead variant in this locus (Table 3 and Additional file 3: Table S2).

**Table 3:** Results for the 42 independent lead SNPs identified in the MTAG-IBS analysis

Locus	Lead SNP	CHR	A1/A2	BP	Cross-trait analysis			FRQ	Nearest Gene	Functional category	Overlap with original GWAS IBS	Overlap with previous GWAs on psychiatric traits	Overlap with previous GWAs	CADD	RDB
					Beta	SE	P								
1	rs301806	1	T/C	8482078	− 0.009	0.002	1.67E-09	0.58	*RERE*	Intronic	*NO*	*Neuroticism*	*Known*	0.117	4
2	rs11206127	1	A/G	53713549	− 0.009	0.002	1.42E-08	0.43	*LRP8*	Intronic	*NO*	*No*	*Novel*	0.128	6
3	rs12755507	1	T/C	176164865	0.01	0.002	8.03E-10	0.625	*RFWD2*	Intronic	*NO*	*Depression*	*Known*	6.038	4
4	rs113198479	1	A/G	191347803	− 0.02	0.004	2.48E-08	0.953	*RP11-309H21.2*	Intergenic	*NO*	*No*	*Novel*	1.241	6
5	rs72740550	1	A/G	197342380	− 0.011	0.002	6.02E-09	0.219	*CRB1*	Intronic	*NO*	*Neuroticism & depression*	*Known*	5.063	7
6	rs115962846	2	A/G	58967058	− 0.015	0.003	3.68E-08	0.912	*LINC01122*	ncRNA_intronic	*NO*	*Neuroticism*	*Known*	2.103	7
7	rs28496790	2	A/C	161950047	0.01	0.002	3.70E-09	0.708	*AC009313.1*	Intergenic	*NO*	*No*	*Novel*	6.027	5
8	rs138218528	2	T/C	212676884	0.009	0.002	2.84E-08	0.667	*ERBB4*	Intronic	*NO*	*Neuroticism & depression*	*Known*	8.481	6
9	rs62246276	3	T/G	9445173	− 0.011	0.002	2.28E-08	0.179	*SETD5*	Intronic	*NO*	*No*	*Novel*	1.944	5
10	rs67416405	3	T/C	85539234	− 0.009	0.002	8.27E-09	0.353	*CADM2*	Intronic	*YES*	*No*	*Known*	3.769	6
11	rs1729951	3	T/G	136500733	− 0.009	0.002	9.01E-09	0.389	*RP11-102M11.2*	Intergenic	*NO*	*Neuroticism*	*Known*	0.078	NA
12	rs1442129	4	A/G	90849446	− 0.009	0.002	1.22E-08	0.453	*MMRN1*	Intronic	*NO*	*No*	*Novel*	5.378	NA
13	rs77087420	4	A/G	123122856	0.018	0.003	2.64E-08	0.945	*KIAA1109*	Intronic	*NO*	*No*	*Novel*	4.579	7
14	rs12513440	5	A/G	7259853	0.01	0.002	2.73E-08	0.243	*RP11-404K5.3*	Intergenic	*NO*	*No*	*Novel*	0.327	5
15	rs3099439	5	T/C	87545318	− 0.011	0.002	1.14E-12	0.539	*TMEM161B*	Intronic	*NO*	*Depression*	*Known*	1.562	NA
16	rs4481363	5	A/C	164474719	0.009	0.001	1.01E-09	0.524	*CTC-340A15.2*	ncRNA_intronic	*NO*	*Neuroticism & depression*	*Known*	6.522	6
16	rs180928232	5	A/G	166185949	− 0.012	0.002	4.46E-08	0.149	*CTB-7E3.1*	Intergenic	*NO*	*Neuroticism*	*Known*	2.692	6
17	rs200977	6	T/C	27854301	0.015	0.002	1.04E-11	0.873	*HIST1H3J*	Intergenic	*NO*	*Neuroticism & depression*	*Known*	1.251	NA
17	rs2534664	6	A/G	31469591	0.01	0.002	2.63E-10	0.456	*MICB*	Intronic	*NO*	*Depression*	*Known*	3.484	NA
17	rs1144708	6	T/C	31710020	− 0.01	0.002	7.49E-10	0.357	*MSH5:MSH5-SAPCD1*	Intronic	*YES*	*No*	*Known*	0.372	6
18	rs12374612	6	T/C	100955752	0.009	0.001	1.02E-08	0.478	*ASCC3*	Downstream	*NO*	*Neuroticism*	*Known*	0.29	6
19	rs2189246	7	A/G	82444372	0.01	0.001	1.98E-10	0.523	*PCLO*	Intronic	*NO*	*Depression*	*Known*	1.139	7
20	rs6956352	7	A/G	109131367	0.009	0.002	1.64E-08	0.458	*AC073071.1*	Intergenic	*NO*	*Depression*	*Known*	9.195	7
21	rs4726814	7	T/C	146691924	− 0.01	0.002	1.30E-08	0.275	*CNTNAP2*	Intronic	*NO*	*No*	*Novel*	1.37	7
22	rs4478545	8	A/G	94672542	− 0.01	0.002	4.77E-09	0.285	*LINC00535*	ncRNA_intronic	*NO*	*No*	*Novel*	1.326	6
23	rs3793577	9	A/G	23737627	− 0.01	0.002	3.46E-10	0.463	*ELAVL2*	Intronic	*NO*	*Neuroticism*	*Known*	19.76	5
24	rs4744242	9	T/G	96236711	− 0.011	0.002	8.68E-13	0.336	*FAM120A*	Intronic	*YES*	*Neuroticism*	*Known*	2.858	6
25	rs10123941	9	T/C	120518162	− 0.01	0.002	3.96E-09	0.727	*snoZ13_snr52*	Intergenic	*NO*	*Neuroticism*	*Known*	1.108	6
26	rs6584631	10	T/C	106656137	− 0.01	0.002	7.23E-09	0.244	*SORCS3*	Intronic	*NO*	*Depression*	*Known*	0.167	4
27	rs4937872	11	A/G	112827715	− 0.012	0.002	7.15E-15	0.589	*RP11-629G13.1*	Intergenic	*YES*	*Neuroticism*	*Known*	0.044	6
28	rs9530139	13	T/C	31847324	− 0.011	0.002	2.11E-08	0.194	*B3GALTL*	Intronic	*NO*	*Depression*	*Known*	0.529	6
29	rs9597797	13	T/G	59183795	− 0.01	0.002	1.42E-09	0.251	*CTAGE16P*	Intergenic	*NO*	*Neuroticism*	*Known*	0.278	7
30	rs2121708	14	A/G	42146572	− 0.009	0.001	8.26E-10	0.517	*LRFN5*	Intronic	*NO*	*Depression*	*Known*	0.043	NA
31	rs35641442	14	A/G	75207263	0.009	0.002	6.65E-09	0.459	*FCF1*	Intergenic	*NO*	*Neuroticism & depression*	*Known*	11.4	7
32	rs1862743	16	A/C	60743834	− 0.009	0.001	1.08E-08	0.492	*GNPATP*	Intergenic	*NO*	*No*	*Novel*	1.06	6
33	rs11877758	18	T/G	35138110	− .,012	0.002	1.28E-13	0,692	*CELF4*	Intronic	*NO*	*Neuroticism & depression*	*Known*	2.718	7
33	rs2978362	18	T/C	32959397	− 0.008	0.001	2.65E-08	0.527	*ZNF396*	Intergenic	*NO*	*Depression*	*Known*	1.024	NA
34	rs12958048	18	A/G	53101598	0.01	0.002	4.76E-11	0,333	*TCF4*	Intronic	*NO*	*Neuroticism*	*Known*	2,08	5
34	rs17410557	18	T/C	50776391	− .,009	0.002	1.13E-08	0,606	*DCC*	Intronic	*NO*	*Neuroticism & depression*	*Known*	4.502	7
35	rs2111530	19	A/G	31891006	− 0.009	0.002	9.47E-09	0.602	*AC007796.1*	ncRNA_intronic	*NO*	*No*	*Novel*	17.04	7
36	rs2024568	20	T/C	44732089	0.011	0.002	1.52E-10	0.246	*RPL13P2*	Intergenic	*NO*	*Neuroticism & depression*	*Known*	0.149	6
37	rs11090039	22	A/G	41496800	0.012	0.002	2.87E-13	0.284	*EP300*	Intronic	*NO*	*Neuroticism*	*Known*	9.707	5

Overlap with previous GWAS was examined by identifying genome-wide significant SNPs within ± 5000 kb in the MTAG genome-wide significant for IBS and original GWAS genome-wide significant SNPs for each trait (i.e. neuroticism, depression and anxiety). If there were overlapping SNPs within this distance, they were considered independent signal if r^2^ > 0.2. The independent signals identified (indicated as novel) were further confirmed using conditonal analysis

*CHR* chromosome; *A1* effect allele with respect to the Beta; *A2* alternate allele; *BP* base pair position Genome Reference Consortium Human Build 37 (GRCh37); *SE* standard error; *FRQ* frequency of the A1; *CADD* Combined Annotation Dependent Depletion score; *RDB* RegulomeDB score

Comparing these results with the ones originally described for IBS [18], 38 out of the 42 SNPs identified herein were novel for IBS and all of them showed consistent direction of the association (Fig. 1a and Additional file 3: Table S3). Of them, 11 were not previously associated with neuroticism, depression or anxiety (Fig. 1d). The remaining signals, 27 in total, were novel associated SNPs for IBS but previously reported for neuroticism and/or depression (Table 3, Fig. 1d) and overall showed consistent direction of association with that reported in the original studies (Fig. 1a). Of the six SNPs previously identified in IBS [18], four of them, on chromosome 3, 6, 9 and 11, were among the significant SNPs for IBS in the current study and the two additional ones, in chromosome 13, showed suggestive evidence of association (*P* < 5E-07; Table 3). Among top findings, we found lead SNPs nearby genes involved in transcriptional regulation, including non-coding RNAs (*RP11*-*629G13.1* and *MSH5-SAPCD1*), RNA splicing (*CELF4*), chromatin remodeling (*EP300* and *HIST1H3J*), mRNA transport (*FAM120A*) or nucleic acid binding (*TCF4* and *ELAVL2*), as well as in brain development (*TMEM161B*) or presynaptic activity (*PCLO*).

### Credible variants and functional annotation

**Fig. 2:**
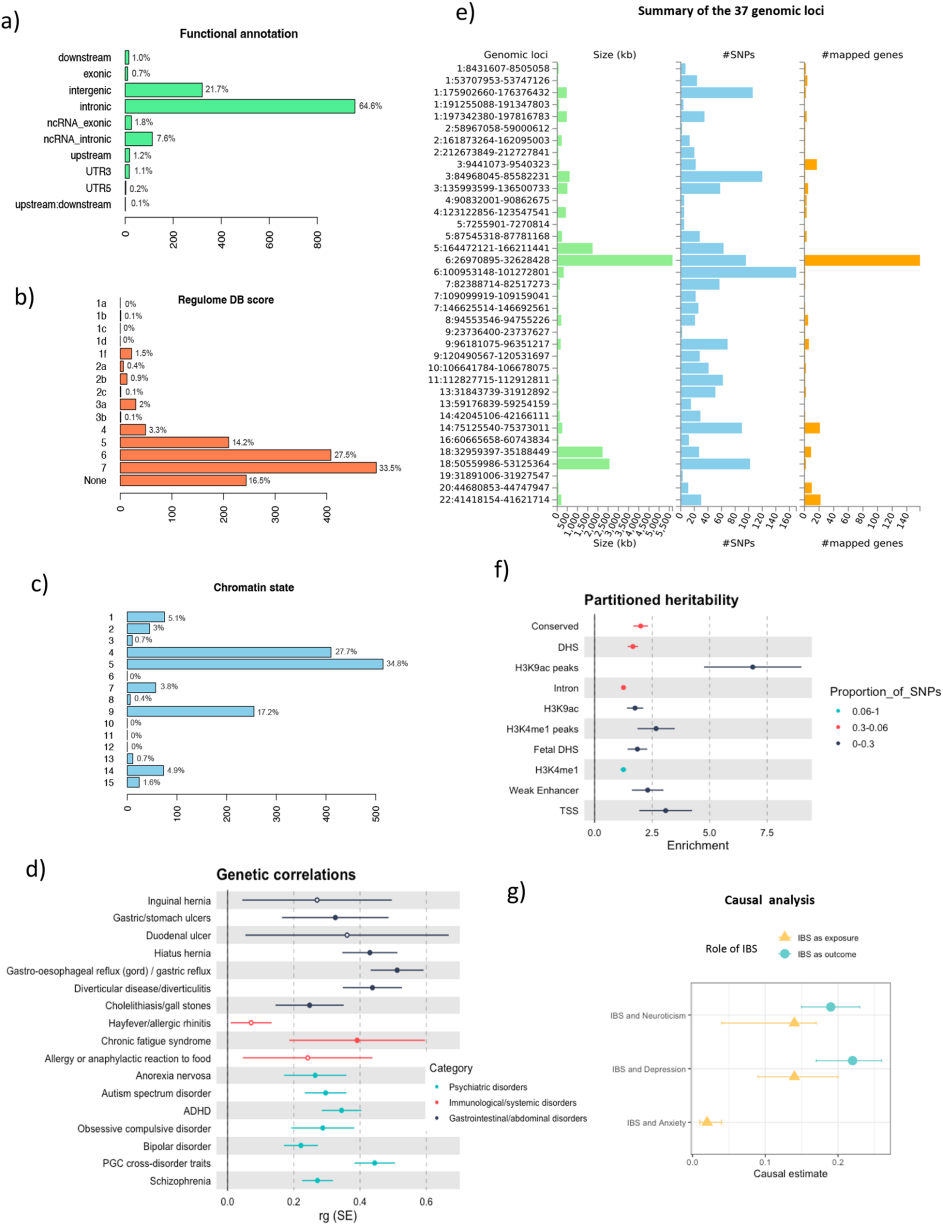
Follow-up analysis of MTAG-IBS results and causal analysis. **a** Functional annotation of the credible variants associated with MTAG-IBS. **b** RegulomeDB scores of the credible variants associated with MTAG-IBS. Low scores indicate increasing likelihood of having regulatory function. **c** Distribution of the credible variants associated with MTAG-IBS across 15 categories of minimum chromatin state. Lower state indicating higher accessibility and states from 1 to 7 refer to open chromatin states. **d** Genetic correlations (rg) between MTAG-IBS results and 17 phenotypes involving digestive, immunological and psychiatric disorders. Only significant correlations after Bonferroni correction are displayed. **e** Bar graphs depicting the size of the genomic locus (left), number of candidate SNPs in the locus (center) and number of mapped genes in the genomic locus (right). Genomic loci are displayed by “chromosome: start position-end position”. **f** Partitioning of the SNP heritability of the MTAG-IBS results using LD Score regression. Enrichment was calculated by dividing the partial heritability of a category by the proportion of SNPs in that category (proportion indicated by color). Only significant enrichments are displayed. **g** Causal relationships between IBS and neuroticism, depression and anxiety assessed using Causal Analysis Using Summary Effect estimates (CAUSE). Only associations with evidence of causal relationship are displayed

We identified a total of 1,818 Bayesian credible variants in the 37 independent loci for IBS (Additional file 3: Table S4). Their functional annotation revealed over-presentation of SNPs in introns (64.6%), intergenic regions (21.7%) or located in non-coding RNA (9.4%) (Fig. 2 and Additional file 3: Table S5). A total of 75% of the variants within credible sets were located in open chromatin regions (minimum chromatin state ≤ 7), 3% were likely to affect the binding of transcription factors (RegulomeDB scores from 1b to 2c) and 0.05% may be deleterious (Combined Annotation Dependent Depletion (CADD) score > 12.37) (Fig. 2 and Additional file 3: Table S5). Forty-eight variants were previously related by GWAS (*P* < 5E-07) to digestive-related phenotypes (e.g. inflammatory bowel disease, gastroesophageal reflux or gut microbiota relative abundance), lifestyle factors (e.g. alcohol consumption, lifetime smoking, coffee consumption or moderate to vigorous physical activity levels) and brain and neuropsychiatric phenotypes (e.g. neuroticism, depression, anxiety, cognition or brain morphology) (Additional file 3: Table S6). In addition, we found that more that half of the credible variants (n = 953; 52%) were expression quantitative trait loci (eQTL) for at least one gene in one brain area (n = 895; 49%) and/or digestive tissue (n = 690; 38%; Additional file 3: Table S7).

Credible variants were mapped to 289 unique genes (Additional file 3: Table S8 and Additional file 2: Figure S4) that were significantly enriched in genes upregulated during early embryonic brain development (8th post conceptual week; Additional file 2: Figure S5) and in several gene-sets (Additional file 3: Table S9). Among the most significant ones, we found psychiatric disorders (GWAS catalog: autism spectrum disorder or schizophrenia, *P*-adjusted = 5.0E-193), digestive disorders (GWAS catalog: ulcerative colitis, *P*-adjusted = 1.1E-57 and inflammatory bowel disease, *P*-adjusted = 7.1E-40), autoimmune disease (KEGG: Systemic lupus erythematosus, *P*-adjusted = 7.9E-61) and histone deacetylases (Reactome: HDACS deacetylate histones, *P*-adjusted = 3.1E-46) (Additional file 3: Table S9).

### Gene-based and gene-set analyses of MTAG-IBS risk loci

The gene-based analysis identified 76 significant genes, which were associated with expression changes in the cerebellum (*P* = 5.2E-09), frontal cortex (*P* = 9.8E-07), anterior cingulate cortex (*P* = 1.8E-05), basal ganglia nuclei (nucleus accumbens: *P* = 6.9E-05; caudate: *P* = 9.7E-04) and hypothalamus (*P* = 4.3E-04) (Additional file 3: Table S10, Additional file 2: Figure S6–S7) as well as with gene expression during the 21st post conceptual week (*P* = 8.5E-04) (Additional file 2: Figure S7). Among top findings, we found genes with a role in brain development and synaptic function, including *CADM2* and *NCAM1,* previously identified in the latest GWAS on IBS, and also genes involved in transcriptional regulation through mRNA transport or chromatin structure, including *FAM120A*, *PHF2* and different histone coding genes. When we conducted the gene-set analysis we found the *branching morphogenesis of a nerve* pathway significantly associated with IBS (gene-set size = 10 genes; *P* = 1.7E-06) (Additional file 3: Table S11).

### Drug target identification

The enrichment analysis on druggable genes showed enrichment of MTAG-IBS-finemapped credible genes in druggable genes for 21 drugs (Additional file 3: Table S12), being l-lysine (*P* < 2.2E-16), belinostat (*P* = 8.6E-10), s-adenosylmethionine (*P* = 7.0E-09) and allopurinol (*P* = 1.5E-07), the top ones (Additional file 3: Table S12). They also included drugs related to musculo-skeletal system, such as anti-inflammatory and antirheumatic drugs, or related to the nervous system, such as anesthetics and drugs used in opioid dependence (Additional file 3: Table S12).

### Partitioned heritability and genetic correlations

When we partitioned the *h*^*2*^_SNP_ of IBS, we observed significant heritability enrichment in ten functional categories (Fig. 2 and Additional file 3: Table S13), with the strongest enrichment of variants in conserved regions (enrichment = 2.01; *P* = 4.0E-09), DNase I hypersensitive sites (DHSs) regions (enrichment = 1.66; *P* = 9.1E-08) and histone H3 lysine 9 acetylation (H3K9ac) peaks (enrichment = 6.88; *P* = 1.1E-07).

We found significant genetic correlations between IBS and 13 gastrointestinal, immunological or psychiatric disorders using GWAS summary statistics available in the MR-Base database [35], including gastric reflux (rg = 0.51; *P* = 2.6E-36), the cross-disorder GWAS from the PGC involving schizophrenia, bipolar disorder, major depressive disorder, autism spectrum disorders and attention-deficit/hyperactivity disorder (ADHD) (rg = 0.44, *P* = 9.7E-46), diverticulitis (rg = 0.44, *P* = 7.4E-22), hiatus hernia (rg = 0.43; *P* = 4.7E-20) and chronic fatigue syndrome (rg = 0.39, *P* = 2.0E-04), among others (Fig. 2 and Additional file 3: Table S14).

### Causal analysis using summary effect estimates (CAUSE)

CAUSE [36] showed consistent evidence for a causal effect of the genetic liability of IBS on neuroticism (ΔELPD = -3.6, SE = 1.9, *P* = 0.031), depression (ΔELPD = -5.9, SE = 1.8, *P* = 5.4E-03) and anxiety (ΔELPD = -2.9, SE = 1.7, *P* = 0.049). We also found evidence for reverse causality with a causal effect of the genetic liability of neuroticism and depression on IBS (ΔELPD = -7.3, SE = 1.4, *P* = 1.5E-07 and ΔELPD = -6.3, SE = 1.4, *P* = 1.8E-06 respectively) but there was no evidence for a causal relationship when anxiety was considered as exposure and IBS as outcome (Fig. 2, Additional file 3: Table S15a, b and Additional file 2: Figure S8).

## Discussion

In the present study we found extensive genetic sharing between IBS, neuroticism, depression and anxiety, and identified 42 genome-wide significant SNPs for IBS, of which 38 are novel. Our findings confirm the polygenic architecture of the disorder, with more than 12,000 variants explaining 90% of the *h*^*2*^_SNP,_ and represent a great advance over the previously reported six genome-wide associated SNPs [18]. Significant signal enrichment was found in genes showing heightened expression in the brain during early embryonic development and playing prominent roles in mental and digestive disorders, autoimmune diseases and transcription regulation.

Our results confirm a role on IBS of genes involved in brain development and synaptic function as well as genes previously associated with psychiatric conditions [18]. We detected 27 SNPs for IBS also associated with at least one of the three mental conditions under study, and found evidence supporting that IBS and neuroticism, which is genetically correlated with many psychiatric disorders [39], share a considerable proportion of their genetic background. The widespread common genetic risk sharing with mental conditions was further supported by the positive genetic correlation found between IBS and many psychiatric disorders (i.e. schizophrenia, ADHD, autism or depression) and by the IBS associated variants being located within genes significantly expressed in the brain. These results are in agreement with the higher burden of mental disorders often co-existing in IBS patients, add further evidence of substantial pleiotropy of contributing loci and underscore that genetic influences on IBS may transcend diagnostic boundaries.

Among top findings we identified genes associated with IBS in previous GWAS, such as *CADM2* and *NCAM1,* members of the synaptic cell adhesion molecules that play a role in synapse organization and plasticity [40, 41]*.* Interestingly, NCAM peptide mimetics have been proven to have both antidepressant and anti-inflammatory effects [42, 43], pointing them as a potential therapeutic target for IBS. Novel loci for IBS include interesting genes previously associated with depression and other mental disorders, such as *RERE*, that regulates retinoic acid signaling during development [44–46], *PCLO,* involved in synaptic vesicle trafficking, *TMEM161B* [47]*,* a brain-expressed transmembrane protein [48], *RBFOX1,* a splicing regulator mainly expressed in neurons, that is one of the most pleiotropic genes among psychiatric disorders [49] or *DRD2,* encoding the dopamine receptor D2R and one of the strongest candidates for psychiatric disorders and traits [50]. Interestingly, several studies in animal models suggested an important role for dopamine signaling both in the development and progression of inflammatory bowel disease [51] and treatment with D2R agonists decreased the severity of ulcerative colitis in mice and rats [52].

Interestingly, three of the identified genome-wide significant SNPs had been tested for association with psychiatric and neurological phenotypes, which contributes to clarify their potential functional role. One of these SNPs is the rs301806 (MTAG-IBS P-value = 1.7E-09) located in chromosome 1 in the *RERE* gene and previously associated with neuroticism. A neuroimaging study of drug-naïve individuals with MDD found that reductions in cortical thickness among patients (n = 47) compared to controls (n = 42) were significantly larger among those with the T/T genotype of this SNP compared to C carriers [53]. Another SNP, the rs4481363 (MTAG-IBS P-value = 1.0E-09) located in chromosome 5 in the *CTC-340A15.2* gene, previously associated with neuroticism and depression, has been examined in a study testing associations between genetic variants associated with subjective well-being and depressive symptoms and these, and metabolic phenotypes in a Chinese elderly sample (n = 1788). However, this SNP did not show association with any of the phenotypes studied [54]. The third SNP is the rs2024568 (MTAG-IBS P-value = 1.5E-10) in chromosome 20 (nearest gene was the *RPL13P2*) previously associated with neuroticism and depression. This variant was identified as likely affecting DNA methylation patterns in multiple sclerosis (MS) in a gene-regulatory network integrating GWAS summary statistics and DNA methylation profiles from 140 cases of MS and 139 controls [55].

We also provide new insights underlying IBS, showing strong evidence of transcriptional regulation mechanisms playing a role in the disorder, including non-coding RNAs and histone modification. The over-representation of credible variants in non-coding regions is a common finding when investigating the genetic basis of complex traits [56]. Although the role of non-coding variants is still unclear, it has been suggested that non-coding variants may impact the phenotype by alteration of regulatory elements such as enhancers, transcription factor binding sites or chromatin state [56]. Indeed, we found 75% of the variants within credible sets were located in open chromatin regions (minimum chromatin state ≤ 7), 3% were likely to affect the binding of transcription factors (RegulomeDB scores from 1b to 2c) and 0.05% may be deleterious (CADD score > 12.37). These results point towards a potential role for IBS associated non-coding variants in gene regulation. More specifically, we found genes encoding histones and histone modifying enzymes among top findings, and enrichment of IBS associations in histone acetylation and methylation peaks and in target genes for the histone deacetylase inhibitor belinostat [57]. These findings are in agreement with previous results involving chromatin modifications in maintenance of anxiety behavior and nociception and in visceral hypersensitivity induced by early-life stress [58, 59]. Additionally, top findings also include non-coding RNAs, an epigenetic mechanism that has been involved in regulation of genes related with visceral pain response and intestinal permeability [60–62]. These results add additional evidence towards the role of epigenetic programming in inflammation, visceral pain as well as in intestinal permeability, sensibility and motility in both humans and animal models of IBS [58, 59, 63, 64].

Despite many of the findings pointing out neurobiological processes and mental disorders, we also detected links between IBS and gastrointestinal-related phenotypes. Fine mapping showed that 38% of the credible variants were eQTLs for at least one digestive tissue and that credible sets were located in genes enriched in different digestive disorders, including ulcerative colitis and inflammatory bowel disease. In addition, positive genetic correlations were found between IBS and gastric reflux, diverticulitis, hiatus hernia, cholelithiasis/gallstones and gastric/stomach ulcers, among others, which adds evidence on the overlap between the genetic risk for IBS and for other digestive-related disorders and traits. These findings may reflect the multi-factorial etiology proposed for IBS involving psychological factors, abnormal brain functioning and dysregulation of brain-gut interactions [15, 65–67], as previously proposed in different psychiatric disorders such as depression [68].

IBS-associated signals were also enriched in target genes of relevant drugs, including l-lysine or S-adenosylmethionine. L-lysine acts as partial serotonin 5-HT4 receptor antagonist and inhibits serotonin-mediated intestinal pathologies in rats, including anxiety and stress-induced fecal excretion and severity of diarrhea [69]. Interestingly, l-lysine, and other 5-HT4 receptor antagonists, are promising targets for the treatment of diarrhea-predominant IBS [70, 71] and may aminorate serotonin disturbances in gut and brain that account for part of intestinal and mental disorders [69]. Additional drugs of interest include S-adenosylmethionine, involved in neurotransmission signaling that has a putative antidepressant effect [72, 73] or allopurinol that improves inflammatory bowel disease clinical outcomes [74], among others.

Despite the high prevalence of psychiatric comorbidities reported in patients with IBS, particularly anxiety and depression, a clear temporal relationship between them has not been well established. We found evidence for a bidirectional causal effect between IBS and neuroticism or depression when accounting for correlated pleiotropy, which strengthens previous evidence [18]. In addition, we found evidence for a causal effect of the genetic liability of IBS on anxiety. These findings support that IBS increases the risk of subsequent depressive and anxiety disorders described in longitudinal study designs [75] and also previous evidence supporting that prior depression raises the risk of developing IBS [76, 77]. We found, however, no evidence for a causal effect of the genetic liability of anxiety on IBS when accounting for correlated pleiotropy, in line with previous results [18]. Although the sample size for anxiety was more limited and these results may also reflect lack of statistical power. Long term follow-up studies as well as larger datasets and sensitivity analyses are required to confirm the robustness of these results and to better understand the temporal relationship between IBS and comorbid mental conditions.

A major strength of our study is the substantial larger sample size compared with previous studies. By conducting meta-analysis of GWAS summary statistics for IBS and comorbid mental conditions with MTAG we increased the effective sample size from 486,601 in the original IBS dataset to 887,490 individuals and the number of IBS genome-wide significant associated SNPs from six in the single-trait analysis to 42. Thirty-eight of them were novel for IBS and 11 were not associated with any of the mental conditions under study, which highlight that MTAG combining GWAS on IBS and mental conditions is a robust strategy to identify trait specific genetic associations. In addition, four of the previously six identified SNPs were also significant in the present study [18]. Even though two identified SNPs demonstrated less association here, their associations were still suggestive (*P* < 5E-07) and in concordance in the direction of the effect with the original GWAS study on IBS, which supports validity of the findings across studies.

The study, however, should be considered in the context of some limitations: (i) We did not account for phenotypic overlap and cannot discard that comorbid conditions may have biased the observed results. Also, IBS is considered a highly heterogenous disorder with pathophysiological differences observed among clinical subtypes, between genders, and across age groups and geographic locations [1]. Accounting for such factors may contribute to better characterize the disorder, capture its genetic background and identify overlap with other comorbid disorders that may impact on IBS risk, prognosis and clinical outcome [6]; (ii) Despite the strong genetic correlation between IBS and the three mental conditions under study, MiXeR was unable to assess the genetic overlap between IBS, depression and anxiety probably due to the high polygenicity and low SNP heritability estimates for these traits (0.083 and 0.099, respectively) and the limited sample size of the original GWAS on anxiety. We cannot discard, either, that due to lack of power we did not detect IBS signals previously reported for anxiety in the original GWAS or evidence for anxiety increasing the risk for IBS in the causality analyses; (iii) gene-based analyses may be inflated as suggested by the lambda over 1, although given the increased power of gene-based over single SNP analyses and the lack of residual stratification or confounding inflation in the MTAG–IBS results, this inflation may just reflect high polygenicity; (iv) Combining GWAS that differ a great deal in power may lead to inflation of FDR, according to MTAG authors [24]. In this study we combined GWAS with different sample sizes, however their mean chi-squared was similar and accordingly the max-FDR estimated in our IBS analysis was 0.02, which suggested no inflation of our results. Moreover, despite increasing considerably the effective sample size for IBS through the addition of multiple mental conditions, a number of outcomes were gastrointestinal-related phenotypes, which further supports this approach.

In summary, we identified novel risk loci for IBS, reveal new insights of its polygenic architecture and extended previous knowledge on the genetic overlap and causal relationships between IBS, neuroticism, depression and anxiety. Overall, we advance our understanding of the biological mechanisms underlying IBS, highlighted candidate genes related to brain development and function as well as transcriptional regulation and provide insight into the association between IBS and comorbid mental disorders.

## Supplementary Information

**Additional file 1.** Supplementary materials.

**Additional file 2: Figure S1.** MiXeR results for IBS and neuroticism. A) Venn diagram depicting the estimated number of trait-influencing variants shared (gray) between IBS and neuroticism. Unique variants for each trait are depicted in blue for IBS and orange for neuroticism. The number of trait-influencing variants in thousands is shown, with the standard error in thousands provided in parentheses. The size of the circles reflects the polygenicity of each phenotype, with larger circles corresponding to greater polygenicity. The estimated genetic correlation (r_g_) is shown in the bar. Red color indicates positive genetic correlation. B) and C) depict conditional Q–Q plots of observed versus expected −log10 p-values in the primary trait as a function of significance of association with a secondary trait at the level of p ≤ 0.1 (orange lines), p ≤ 0.01 (green lines), p ≤ 0.001 (red lines). Blue line indicates all SNPs. Dotted lines in blue, orange, green, and red indicate model predictions for each stratum. Black dotted line is the expected Q–Q plot under null (no SNPs associated with the phenotype). D) Log-likelihood curves highlighting the goodness of model fit. The minimum point indicates the best-fitting model estimate of the number of influencing variants shared between two traits (Supplementary Table 1). **Figure S2.** LD Score regression plot with the MTAG-IBS results. Each point represents an LD score quantile. The x-axis represents the mean LD score for the variants included in the quantile and the y-axis represents the mean χ2 of variants in that quantile. The black line is the LD score regression line fitted by a linear regression model with mean χ2 as the outcome variable and mean LD score for each bin as the independent variable (Coefficient=0.011, p=2E-16). **Figure S3. **Regional Plots of the 42 lead SNPs identified in the MTAG-IBS analysis. In red, genes mapped by SNPs in the credible sets based on physical proximity, chromatin interaction and/or eQTLs using FUMA. **Figure S4.** Gene-based test QQ plot. Observed versus expected gene-based test p-values on the-log10 scale are shown. Lambda: 1.6855. **Figure S5.** Enrichment of genes mapped to MTAG-IBS variants with credible sets on Differentially Expressed Genes (DEG) in brain tissue. Results from hypergeometric test evaluating enrichment of the 289 mapped genes by credible variants in DEG in brain tissue representing different brain developmental stages in BrainSpan. Significant enrichment at Bonferroni corrected P-value ≤ 0.05 are coloured in red. **Figure S6.** MAGMA tissue expression analysis using GTEx v.8. Results from MAGMA gene-property analysis between gene-based MTAG-IBS associations and tissue specific gene expression profiles. (A) GTEx v.8 54 tissues. (B) GTEx v.8 30 general tissues. Red bars indicate significant results. **Figure S7.** MAGMA tissue expression analysis using Brainspan. Results from MAGMA gene-property analysis between gene-based MTAG-IBS results and tissue specific gene expression profiles in Brainspan. (A) BrainSpan 29 ages. (B) Brainspan 11 developmental stages. Red bars indicate significant results. **Figure S8.** Scatter plots of the causal analysis. Scatter plots of exposure versus outcome effect sizes for: the sharing model (left) illustrating the pattern induced by a shared factor (correlated pleiotropy, eta) without a causal effect; the causal model (middle) illustrating the pattern induced when including also a causal effect (gamma); and the expected log pointwise posterior density (DEPLD) contribution from each variant for each causal relationship tested.

**Additional file 3: Table S1.** a Univariate and bivariate MiXeR output for IBS vs. neuroticism. **Table S2.** Results from association analyses of lead and secondary lead variants conditioned on the lead variant using COJO. For locus with more than two secondary variants, we further check independency of the secondary variants among each other. P-value of the secondary variants in MTAG-IBS and P-value after conditional analysis using COJO are given. The last column in the right indicates whether the secondary variant was considered as an independent signal. **Table S3.** Results in the original GWAS on IBS of the lead SNPs from MTAG-IBS. **Table S4.** Credible variants for each of the 37 independent loci or IBS identified in the cross-trait analysis using MTAG. Variants included herein were identified by all three fine-mapping methods (PAINTOR, CAVIARBF and FINEMAP). The prosterior probability of the variant being causal estimated by PAINTOR, CAVIARBF and FINEMAP is indicated in the last three columns. Chromosome (CHR), base position (BP), and SNP of the index variants. Effect alle (A1) and non-effect allele (A2) with respect to the beta (BETA). The standard error of the beta (SE) and the association P-value. **Table S5.** Functional annotation of the credible variants for each of the 42 lead variants identified in the cross-trait analysis of IBS using MTAG. Chromosome (CHR), SNP of the index variants, base position (BP), combined Annotation Dependent Depletion score (CADD), RegulomeDB score (RDB) and chromatin state (minChrState) are indicated. **Table S6.** GWAS Catalog results for MTAG-IBS credible set variants associated with other traits in previous GWAS. **Table S7.** Variants in credible sets mapped to genes associated with eQTLs. **Table S8.** Genes mapped to sets of crediable variants in FUMA. **Table S9.** Enrichment of genes mapped to variants in credible sets. **Table S10.** Results of the gene-based asociation analysis on MTAG-IBS using MAGMA. Only gene-wide signficant genes are shown. The p-value for gene-wide significance after Bonferroni correction was 0.05/18,135=2.757x10-6. **Table S11.** Results of the gene-set analysis on MTAG-IBS using MAGMA. **Table S12.** Enrichment analysis on druggable genes for MTAG-IBS genes using data from PharmaKG.Categories according to the Anatomical Therapeutic Chemical (ATC) classification system are provided. **Table S13.** Results of the partitioned heritability analysis using LDSC. **Table S14.** Genetic correlations between MTAG-IBS and 28 phenotypes inlcuding digestive, immnological and psychiatric disorders from MR-Base. **Table S15**. a CAUSE model comparison.

## Data Availability

All data used in the current study is publicly available. Summary statistics for IBS can be download from European Bioinformatics Institute GWAS Catalog (https://www.ebi.ac.uk/gwas/). Summary statistics for neuroticism can be downloaded from https://ctg.cncr.nl/software/summary_statistics/ and http://www.ccace.ed.ac.uk. Summary statistics for depression can be downloaded from https://datashare.ed.ac.uk/handle/10283/3203. Summary statistics for anxiety can be downloaded from http://www.nealelab.is/uk-biobank. Genotype tissue expression (GTEx v8) portal: http://www.gtexportal.org/home/datasets. BRAINEAC: http://www.braineac.org. eQTL catalogue: https://www.ebi.ac.uk/eqtl/Methods/. PsychENCODE: http://resource.psychencode.org. CommonMind Consortium (CMC/CMC): https://www.synapse.org//#!Synapse:syn5585484. WEB-based GEne SeT AnaLysis Toolkit (WebGestAlt): http://www.webgestalt.org. SNP heritability and genetic correlations: https://github.com/bulik/ldsc. MiXeR: https://github.com/precimed/mixer. Conditional analysis: https://yanglab.westlake.edu.cn/software/gcta/#COJO. Multi-Trait Analysis of GWAS (MTAG): https://github.com/omeed-maghzian/mtag). Fine-mapping: https://github.com/mulinlab/CAUSALdb-finemapping-pip. Functional Mapping and Annotation of Genome-Wide Association Studies (FUMA): https://fuma.ctglab.nl/. Partitioned heritability: https://github.com/bulik/ldsc/wiki/Partitioned-Heritability. MR-Base database: https://github.com/MRCIEU/mrbase_casestudies. Causal Analysis Using Summary Effect estimates (CAUSE): https://jean997.github.io/cause/pipeline.html. The use of each software tools has been described in the Methods section. Analysis code and scripts used in the current study are available upon request from the corresponding authors.

## Abbreviations

ADHD: Attention-deficit/hyperactivity disorder
AIC: Akaike information criterion
ATC: Anatomical therapeutic chemical
CAUSE: Causal analysis using summary effect estimates
IBS: Irritable bowel syndrome
FDR: False discovery rate
ELPD: Expected log pointwise posterior density
FUMA: Functional Mapping and Annotation of GWAS
GCTA: Genome-wide complex trait analysis
GWAS: Genome-wide association study
*h*^*2*^_SNP_: SNP heritability
LDSC: Linkage disequilibrium score regression
MAGMA: Generalized gene-set analysis of GWAS data
MTAG: Multi-trait analysis of GWAS

## References

[1] Canavan C, West J, Card T (2014). The epidemiology of irritable bowel syndrome. Clin Epidemiol.

[2] Sperber AD, Bangdiwala SI, Drossman DA, Ghoshal UC, Simren M, Tack J (2021). Worldwide prevalence and burden of functional gastrointestinal disorders, results of rome foundation global study. Gastroenterology.

[3] Yeh H-W, Chien W-C, Chung C-H, Hu J-M, Tzeng N-S (2018). Risk of psychiatric disorders in irritable bowel syndrome-A nationwide, population-based, cohort study. Int J Clin Pract.

[4] Ford AC, Moayyedi P, Chey WD, Harris LA, Lacy BE, Saito YA (2018). American college of gastroenterology monograph on management of irritable bowel syndrome. Am J Gastroenterol.

[5] Zamani M, Alizadeh-Tabari S, Zamani V (2019). Systematic review with meta-analysis: the prevalence of anxiety and depression in patients with irritable bowel syndrome. Aliment Pharmacol Ther.

[6] Lee S, Wu J, Ma YL, Tsang A, Guo WJ, Sung J (2009). Irritable bowel syndrome is strongly associated with generalized anxiety disorder: a community study. Aliment Pharmacol Ther.

[7] Lee C, Doo E, Choi JM, Ho JS, Ryu HS, Lee JY (2017). The Increased Level of Depression and Anxiety in Irritable Bowel Syndrome Patients Compared with Healthy Controls: Systematic Review and Meta-analysis. J Neurogastroenterol Motil..

[8] Fond G, Loundou A, Hamdani N, Boukouaci W, Dargel A, Oliveira J (2014). Anxiety and depression comorbidities in irritable bowel syndrome (IBS): a systematic review and meta-analysis. Eur Arch Psychiatry Clin Neurosci.

[9] Zhang QE, Wang F, Geng Q, Zheng W, Ng CH, Ungvari GS (2018). Depressive symptoms in patients with irritable bowel syndrome: a meta-analysis of comparative studies. Int J Biol Sci.

[10] Hazlett-Stevens H, Craske MG, Mayer EA, Chang L, Naliboff BD (2003). Prevalence of irritable bowel syndrome among university students: the roles of worry, neuroticism, anxiety sensitivity and visceral anxiety. J Psychosom Res.

[11] Lydiard RB (2001). Irritable bowel syndrome, anxiety, and depression: what are the links?. J Clin Psychiatry.

[12] Nisticò V, Rossi RE, D’Arrigo AM, Priori A, Gambini O, Demartini B (2022). Functional neuroimaging in irritable bowel syndrome: a systematic review highlights common brain alterations with functional movement disorders. J Neurogastroenterol Motil.

[13] Saffouri GB, Shields-Cutler RR, Chen J, Yang Y, Lekatz HR, Hale VL (2019). Small intestinal microbial dysbiosis underlies symptoms associated with functional gastrointestinal disorders. Nat Commun.

[14] Aguilera-Lizarraga J, Hussein H, Boeckxstaens GE (2022). Immune activation in irritable bowel syndrome: what is the evidence?. Nat Rev Immunol.

[15] Ford AC, Sperber AD, Corsetti M, Camilleri M (2020). Irritable bowel syndrome. The Lancet.

[16] Smoller JW (2016). The genetics of stress-related disorders: PTSD, depression, and anxiety disorders. Neuropsychopharmacology.

[17] Sullivan PF, Neale MC, Kendler KS (2000). Genetic epidemiology of major depression: review and meta-analysis. Am J Psychiatry.

[18] Eijsbouts C, Zheng T, Kennedy NA, Bonfiglio F, Anderson CA, Moutsianas L (2021). Genome-wide analysis of 53,400 people with irritable bowel syndrome highlights shared genetic pathways with mood and anxiety disorders. Nat Genet.

[19] Wu Y, Murray GK, Byrne EM, Sidorenko J, Visscher PM, Wray NR (2021). GWAS of peptic ulcer disease implicates Helicobacter pylori infection, other gastrointestinal disorders and depression. Nat Commun.

[20] Nagel M, Jansen PR, Stringer S, Watanabe K, De Leeuw CA, Bryois J (2018). Meta-analysis of genome-wide association studies for neuroticism in 449,484 individuals identifies novel genetic loci and pathways. Nat Genet.

[21] Howard DM, Adams MJ, Clarke TK, Hafferty JD, Gibson J, Shirali M (2019). Genome-wide meta-analysis of depression identifies 102 independent variants and highlights the importance of the prefrontal brain regions. Nat Neurosci.

[22] Bulik-Sullivan BK, Loh P-R, Finucane HK, Ripke S, Yang J (2015). LD score regression distinguishes confounding from polygenicity in genome-wide association studies. Nat Genet.

[23] Frei O, Holland D, Smeland OB, Shadrin AA, Fan CC, Maeland S (2019). Bivariate causal mixture model quantifies polygenic overlap between complex traits beyond genetic correlation. Nat Commun.

[24] Turley P, Walters RK, Maghzian O, Okbay A, Lee JJ, Fontana MA (2018). Multi-trait analysis of genome-wide association summary statistics using MTAG. Nat Genet.

[25] Maihofer AX, Choi KW, Coleman JRI, Daskalakis NP, Denckla CA, Ketema E (2022). Enhancing discovery of genetic variants for posttraumatic stress disorder through integration of quantitative phenotypes and trauma exposure information. Biol Psychiatry.

[26] Yang J, Ferreira T, Morris AP, Medland SE, Genetic Investigation of ANthropometric Traits (GIANT) Consortium, DIAbetes Genetics Replication And Meta-analysis (DIAGRAM) Consortium (2012). Conditional and joint multiple-SNP analysis of GWAS summary statistics identifies additional variants influencing complex traits. Nat Genet.

[27] Benner C, Spencer CCA, Havulinna AS, Salomaa V, Ripatti S, Pirinen M (2016). FINEMAP: efficient variable selection using summary data from genome-wide association studies. Bioinformatics.

[28] Greenbaum J, Deng HW (2017). A statistical approach to fine mapping for the identification of potential causal variants related to bone mineral density. J Bone Miner Res.

[29] Chen W, Larrabee BR, Ovsyannikova IG, Kennedy RB, Haralambieva IH, Poland GA (2015). Fine mapping causal variants with an approximate bayesian method using marginal test statistics. Genetics.

[30] Wang J, Huang D, Zhou Y, Yao H, Liu H, Zhai S (2020). CAUSALdb: a database for disease/trait causal variants identified using summary statistics of genome-wide association studies. Nucleic Acids Res.

[31] Watanabe K, Taskesen E, Van Bochoven A, Posthuma D (2017). Functional mapping and annotation of genetic associations with FUMA. Nat Commun.

[32] de Leeuw CA, Mooij JM, Heskes T, Posthuma D (2015). MAGMA: generalized gene-set analysis of GWAS data. PLoS Comput Biol.

[33] Liao Y, Wang J, Jaehnig EJ, Shi Z, Zhang B (2019). WebGestalt 2019: gene set analysis toolkit with revamped UIs and APIs. Nucleic Acids Res.

[34] Finucane HK, Bulik-Sullivan B, Gusev A, Trynka G, Reshef Y, Loh P-R (2015). Partitioning heritability by functional annotation using genome-wide association summary statistics. Nat Genet.

[35] Hemani G, Zheng J, Elsworth B, Wade KH, Haberland V, Baird D (2018). The MR-base platform supports systematic causal inference across the human phenome. Elife.

[36] Morrison J, Knoblauch N, Marcus JH, Stephens M, He X (2020). Mendelian randomization accounting for correlated and uncorrelated pleiotropic effects using genome-wide summary statistics. Nat Genet.

[37] Nagel M, Jansen PR, Stringer S, Watanabe K, De Leeuw CA, Bryois J (2018). Meta-analysis of genome-wide association studies for neuroticism in 449,484 individuals identifies novel genetic loci and pathways. Nat Genet.

[38] Luciano M, Hagenaars SP, Davies G, Hill WD, Clarke TK, Shirali M (2018). Association analysis in over 329,000 individuals identifies 116 independent variants influencing neuroticism. Nat Genet.

[39] Anttila V, Bulik-Sullivan B, Finucane HK, Walters RK, Bras J, Duncan L (2018). Analysis of shared heritability in common disorders of the brain. Science.

[40] Boutwell B, Hinds D, Agee M, Alipanahi B, Auton A, Bell RK (2017). Replication and characterization of CADM2 and MSRA genes on human behavior. Heliyon.

[41] Lüthi A, Laurent JP, Figurovt A, Mullert D, Schachnert M (1994). Hippocampal long-term potentiation and neural cell adhesion molecules L1 and NCAM. Nature.

[42] Downer EJ, Cowley TR, Lyons A, Mills KHG, Berezin V, Bock E (2010). A novel anti-inflammatory role of NCAM-derived mimetic peptide. FGL Neurobiol Aging.

[43] Turner CA, Lyons DM, Buckmaster CL, Aurbach EL, Watson SJ, Schatzberg AF (2018). Neural cell adhesion molecule peptide mimetics modulate emotionality: pharmacokinetic and behavioral studies in rats and non-human primates. Neuropsychopharmacology.

[44] Fregeau B, Kim BJ, Hernández-García A, Jordan VK, Cho MT, Schnur RE (2016). De Novo mutations of RERE cause a genetic syndrome with features that overlap those associated with proximal 1p36 deletions. Am J Hum Genet.

[45] Jordan VK, Fregeau B, Ge X, Giordano J, Wapner RJ, Balci TB (2018). Genotype–phenotype correlations in individuals with pathogenic RERE variants. Hum Mutat.

[46] Wray NR, Ripke S, Mattheisen M, Trzaskowski M, Byrne EM, Abdellaoui A (2018). Genome-wide association analyses identify 44 risk variants and refine the genetic architecture of major depression. Nat Genet.

[47] Mbarek H, Milaneschi Y, Hottenga JJ, Ligthart L, De Geus EJC, Ehli EA (2017). Genome-wide significance for PCLO as a gene for Major depressive disorder. Twin Res Hum Genet.

[48] Hyde CL, Nagle MW, Tian C, Chen X, Paciga SA, Wendland JR (2016). Identification of 15 genetic loci associated with risk of major depression in individuals of European descent. Nat Genet.

[49] Lee PH, Anttila V, Won H, Feng YCA, Rosenthal J, Zhu Z (2019). Genomic relationships, novel Loci, and pleiotropic mechanisms across eight psychiatric disorders. Cell.

[50] Ayano G (2016). Dopamine: receptors, functions, synthesis pathways, locations and mental disorders review of literatures. J Ment Disord Treat.

[51] Kurnik-łucka M, Pasieka P, Łączak P, Wojnarski M, Jurczyk M, Gil K (2021). Gastrointestinal dopamine in inflammatory bowel diseases: a systematic review. Int J Mol Sci.

[52] Tolstanova G, Deng X, Ahluwalia A, Paunovic B, Prysiazhniuk A, Ostapchenko L (2015). Role of dopamine and D2 dopamine receptor in the pathogenesis of inflammatory bowel disease. Dig Dis Sci.

[53] Katsuki A, Kakeda S, Watanabe K, Igata R, Otsuka Y, Kishi T (2019). A single-nucleotide polymorphism influences brain morphology in drug-naïve patients with major depressive disorder. Neuropsychiatr Dis Treat.

[54] Wang Y, Ma T, Zhu YS, Chu XF, Yao S, Wang HF (2017). The KSR2-rs7973260 polymorphism is associated with metabolic phenotypes, but not psychological phenotypes, in Chinese elders. Genet Test Mol Biomarkers.

[55] Manuel AM, Dai Y, Jia P, Freeman LA, Zhao Z (2023). A gene regulatory network approach harmonizes genetic and epigenetic signals and reveals repurposable drug candidates for multiple sclerosis. Hum Mol Genet.

[56] Schipper M, Posthuma D (2022). Demystifying non-coding GWAS variants: an overview of computational tools and methods. Hum Mol Genet.

[57] Beck HC, Nielsen EC, Matthiesen R, Jensen LH, Sehested M, Finn P (2006). Quantitative proteomic analysis of post-translational modifications of human histones. Mol Cell Proteomics.

[58] Aguirre JE, Winston JH, Sarna SK (2017). Neonatal immune challenge followed by adult immune challenge induces epigenetic-susceptibility to aggravated visceral hypersensitivity. Neurogastroenterol Motil.

[59] Moloney RD, Stilling RM, Dinan TG, Cryan JF (2015). Early-life stress-induced visceral hypersensitivity and anxiety behavior is reversed by histone deacetylase inhibition. Neurogastroenterol Motil.

[60] Vicario M, Martínez C, Santos J (2010). Role of microRNA in IBS with increased gut permeability. Gut.

[61] Martínez C, Rodinõ-Janeiro BK, Lobo B, Stanifer ML, Klaus B, Granzow M (2017). Original article: miR-16 and miR-125b are involved in barrier function dysregulation through the modulation of claudin-2 and cingulin expression in the jejunum in IBS with diarrhoea. Gut.

[62] Wohlfarth C, Schmitteckert S, Härtle JD, Houghton LA, Dweep H, Fortea M (2017). miR-16 and miR-103 impact 5-HT4 receptor signalling and correlate with symptom profile in irritable bowel syndrome. Sci Rep.

[63] Hong S, Zheng G, Wiley JW (2015). Epigenetic regulation of genes that modulate chronic stress-induced visceral pain in the peripheral nervous system. Gastroenterology.

[64] Tran L, Schulkin J, Ligon CO, Greenwood-Van MB (2015). Epigenetic modulation of chronic anxiety and pain by histone deacetylation. Mol Psychiatry.

[65] Staudacher HM, Mikocka-Walus A, Ford AC (2021). Common mental disorders in irritable bowel syndrome: pathophysiology, management, and considerations for future randomised controlled trials. Lancet Gastroenterol Hepatol.

[66] Carabotti M, Scirocco A, Maselli MA, Severi C (2015). The gut-brain axis: interactions between enteric microbiota, central and enteric nervous systems. Ann Gastroenterol.

[67] Fadgyas-Stanculete M, Buga A-M, Popa-Wagner A, Dumitrascu DL (2014). The relationship between irritable bowel syndrome and psychiatric disorders: from molecular changes to clinical manifestations. J Mol Psychiatry.

[68] Evrensel A, Ceylan ME (2015). The gut-brain axis: the missing link in depression. Clin Psychopharmacol Neurosci.

[69] Smriga M, Torii K (2003). L-Lysine acts like a partial serotonin receptor 4 antagonist and inhibits serotonin-mediated intestinal pathologies and anxiety in rats. Proc Natl Acad Sci USA.

[70] Bharucha AE, Zinsmeister AR, Camilleri M, Haydock S, Ferber I, Burton D (2000). Effects of a serotonin 5-HT4 receptor antagonist SB-207266 on gastrointestinal motor and sensory function in humans. Gut.

[71] Tam FS-F, Hillier K, Bunce KT (1994). Characterization of the 5-hydroxytryptamine receptor type involved in inhibition of spontaneous activity of human isolated colonic circular muscle. Br J Pharmacol.

[72] Papakostas GI, Mischoulon D, Shyu I, Alpert JE, Fava M (2010). S-adenosyl methionine (SAMe) augmentation of serotonin reuptake inhibitors for antidepressant nonresponders with major depressive disorder: a double-blind, randomized clinical trial. Am J Psychiatry.

[73] Sharma A, Gerbarg P, Bottiglieri T, Massoumi L, Carpenter LL, Lavretsky H (2017). S-adenosylmethionine (SAMe) for neuropsychiatric disorders: a clinician-oriented review of research. J Clin Psychiatry.

[74] Moreau B, Clement P, Theoret Y, Seidman EG (2017). Allopurinol in combination with thiopurine induces mucosal healing and improves clinical and metabolic outcomes in IBD. Therap Adv Gastroenterol.

[75] Lee YT, Hu LY, Shen CC, Huang MW, Tsai SJ, Yang AC (2015). Risk of psychiatric disorders following irritable bowel syndrome: a nationwide population-based cohort study. PLoS ONE.

[76] Sibelli A, Chalder T, Everitt H, Workman P, Windgassen S, Moss-Morris R (2016). A systematic review with meta-analysis of the role of anxiety and depression in irritable bowel syndrome onset. Psychol Med.

[77] Goodwin L, White PD, Hotopf M, Stansfeld SA, Clark C (2013). Life course study of the etiology of self-reported irritable bowel syndrome in the 1958 British birth cohort. Psychosom Med.

